# Redox correlation in muscle lengthening and immune response in eccentric exercise

**DOI:** 10.1371/journal.pone.0208799

**Published:** 2018-12-27

**Authors:** Feng He, Chia-Chen Chuang, Tingyang Zhou, Qing Jiang, Darlene A. Sedlock, Li Zuo

**Affiliations:** 1 Department of Health and Kinesiology, College of Health and Human Sciences, Purdue University, West Lafayette, IN, United States of America; 2 Department of Kinesiology, California State University-Chico, Chico, CA, United States of America; 3 Radiologic Sciences and Respiratory Therapy Division, School of Health and Rehabilitation Sciences, The Ohio State University College of Medicine, The Ohio State University Wexner Medical Center, Columbus, OH, United States of America; 4 Interdisciplinary Biophysics Graduate Program, The Ohio State University, Columbus, OH, United States of America; 5 Department of Nutrition Science, College of Health and Human Science, Purdue University, West Lafayette, IN, United States of America; 6 Molecular Physiology and Biophysics Laboratory, College of Arts and Sciences, University of Maine, Presque Isle, ME, United States of America; University of California, Davis, UNITED STATES

## Abstract

This study was designed to examine the potential involvement of reactive oxygen species in skeletal muscle dysfunction linked with stretching in a mouse model and to explore the effects of combined antioxidant intake on peripheral leukocyte apoptosis following eccentrically-biased downhill runs in human subjects. In the mouse model, diaphragmatic muscle was stretched by 30% of its optimal length, followed by 5-min contraction. Muscle function and extracellular reactive oxygen species release was measured *ex vivo*. In human models, participants performed two trials of downhill running either with or without antioxidant supplementation, followed by apoptotic assay of inflammatory cells in the blood. The results showed that stretch led to decreased muscle function and prominent ROS increase during muscle contraction. In human models, we observed an elevation in circulating leukocyte apoptosis 24–48 hours following acute downhill runs. However, there is an attenuated leukocyte apoptosis following the second bout of downhill run. Interestingly, the combination of ascorbic acid (vitamin C) and α-tocopherol (vitamin E) supplementation attenuated the decrease in B-cell lymphoma 2 (Bcl-2) at 24 hours following acute downhill running. These data collectively suggest that significant ROS formation can be induced by muscle-lengthening associated with eccentric exercise, which is accompanied by compromised muscle function. The combination of antioxidants supplementation appears to have a protective role via the attenuation of decrease in anti-apoptotic protein.

## Introduction

Eccentric exercise-induced muscle damage is characterized by decreased muscle contractile force, muscle stiffness, swelling, delayed-onset muscle soreness, and histological alterations involving myofilament, extracellular matrix (ECM), and epimysium [1]. Active muscle lengthening, as seen during eccentric exercise, is generally associated with muscle damage due to non-uniform stretching of the sarcomeres [2]. Stretched sarcomeres may become weakened and fail to correctly re-interdigitate the thick and thin filaments when the muscle relaxes. Since sarcomeres are arranged in series, the damage induced by extended sarcomeres can propagate longitudinally, collectively resulting in reduced production of force during the next contraction [3]. In the myocardium, stretched myocardium has shown to trigger myocyte apoptosis and superoxide (O_2_^•–^) production [4]. Although skeletal muscle exhibits a greater adaptive ability to stretching than myocardium through sarcomerogenesis and hypertrophy [2, 5], the disruption of sarcomere stability inadvertently impairs muscle force development. It is postulated that muscle damage resulting from eccentric exercise is associated with disrupted sarcomeres and subsequent excitation-contraction coupling dysfunction. Indeed, sarcomere lengthening is considered an initiative event for a muscle-damaging processes during eccentric contractions [6].

In addition to mechanical stress-induced muscle damage, eccentric exercise has been shown to elicit greater leukocyte apoptosis and oxidative stress compared to concentric exercise, both of which may contribute to post-exercise immune suppression, in comparison to concentric exercise [7, 8]. It is well documented that exhaustive exercise is associated with a decrease in circulating immune cells, likely due to leukocyte apoptosis [9, 10]. Syu *et al*. reported that acute vigorous exercise accelerates neutrophil apoptosis and exacerbates oxidative stress, whereas chronic moderate exercise can attenuate neutrophil apoptosis by altering the redox status of neutrophils toward a more reduced state [11]. Both *in vitro* and *in vivo* studies indicate that excessive reactive oxygen species (ROS) limit cell lifespans by activating death receptor signaling or dysregulating the mitochondrial transmembrane potential [12]. Additionally, ROS can directly damage DNA and proteins, promoting muscle fatigue [13]. Moreover, ROS produced in skeletal muscle have been shown to play a crucial role in regulating mitogen-activated protein kinases (MAPKs) pathways [14]. Marked activation of MAPKs including p38 and extracellular signal-regulated kinase 1/2 (ERK1/2) was observed 30 min after eccentric exercise as compared to concentric exercise, suggesting that a greater level of ROS are induced by eccentric exercise during muscle contraction [15].

The correlation of muscle function alteration and related leukocyte apoptosis following stretching or eccentric contractions remains to be further elucidated. Emerging evidence supports a major role of ROS and their elevation being implicated in eccentric exercise-induced injury [3, 16, 17]. Previous studies have reported that a second bout of eccentric exercise which was performed within several weeks from the first exercise bout is linked with a marked attenuation of muscle functional decline [18]. This protective effect has been attributed to neural adaptations, an increase in sarcomere numbers in series, ECM remodeling, and an altered inflammatory response [1]. Interestingly, oxidative stress and leukocyte apoptosis are significantly lowered after the second bout of eccentric exercise [7, 19]. In addition, antioxidant supplementation has shown to not only attenuate eccentrically-biased exercise-induced muscle damage/soreness but also enhance the protective effect following the second bout of eccentric exercise [20]. This adaptation blunts the detrimental effects induced by unaccustomed eccentric exercise such as muscle tenderness, pain, and strength loss, which may occur in response to the high mechanical stress of eccentric contraction [21, 22]. Thus, it is essential to explore the redox interaction in muscle force development and immune response during unaccustomed eccentric exercise. If increased oxidative stress promotes leukocyte apoptosis, antioxidant supplementation should suppress leukocyte apoptosis after acute strenuous exercise by attenuating the increase in ROS. Stretching of sarcomere is considered as the major mechanism underlying muscle damage associated with eccentric exercise [1]. However, the exact mechanism of ROS production during muscle stretch has not been fully elucidated. Therefore, in this study, we first examined the extracellular ROS formation and muscle function in stretched mouse diaphragm during contraction. Additionally, we explored the effects of antioxidant supplementation on exercise-induced leukocyte apoptosis and during repeated bouts of eccentrically-biased exercise (i.e., downhill running) with human subjects. Understanding the relevance of ROS will provide insights to further study in eccentric exercise and its related injuries.

## Materials and methods

### Animal model: Muscle lengthening experiment using mouse model

#### Mouse diaphragm preparation and stretching protocol

This study was carried out in strict accordance with the recommendations in the Guide for the Care and Use of Laboratory Animals of the National Institutes of Health. All experimental procedures involving mice were approved by The Ohio State University Institutional Animal Care and Use Committee (IACUC; Protocol Number: 2013A00000046-R1) and strictly adhered to the guidelines. Male C57BL/6 mice were used in the study. Each mouse was anesthetized with an intraperitoneal injection of ketamine (80 mg/kg) and xylazine (10 mg/kg). The diaphragm muscle was quickly dissected out and cut into two strips (~0.5 cm wide) with a small part of tendon and rib attached in the presence of Ringer’s solution (in mM: 121 NaCl, 5.9 KCl, 1.2 NaH_2_PO_4_, 0.9 Na_2_SO_4_, 2.0 CaCl_2_, 1.0 MgCl_2_, 21 NaHCO_3_, 11.5 glucose; pH 7.4, room temperature). Each strip was mounted in an experimental chamber (model 800MS; Danish Myo Technology, Aarhus, Denmark) by securing the corresponding central tendon to a mobile lever arm designed to adjust muscle length [23]. The chamber was then filled with oxygenated Ringer’s solution containing 20 μM cyt *c*, which is an extracellular O_2_^•–^probe (Sigma, St. Louis, MO) to examine O_2_^•–^released from tissue [24].

Each muscle strip was carefully stretched and the optimal muscle length was determined when the muscle reached its highest twitch force. The muscle length between the tendon and rib was then measured using a ruler (in cm). After determining the optimal muscle length, the muscle strip was further stretched by 30% of its optimal length and equilibrated in oxygenated Ringer’s solution at room temperature for 15–20 min. Each muscle strip was then electrically stimulated by an S48 stimulator (Grass Technologies, West Warwick, RI) with square-wave pulses (70Hz, 250-ms train duration, 0.5-ms pulse duration) for 5 min at 37°C, following the previous contractile protocols [25]. Muscle force (mN/mg) was measured by a stationary force transducer (force range 0 to 1,600 mN) [26]. The tetanic contractions were recorded and converted to digital data by an A-D converter (model ML826; AD instruments, Colorado Springs, CO). LabChart 7.3.1 was employed to analyze the data. Cyt *c* was added to the strip during the 5-min contraction with or without the addition of SOD. Cyt *c* can be reduced by O_2_^•–^to generate an enhanced absorbance at 550 nm, which was measured via a spectrophotometer (Nanodrop 2000, Thermal Scientific, MA, USA). This absorbance is directly correlated to the concentrations of extracellular O_2_^•–^with an extinction coefficient 18.5×10^3^ M^−1^cm^−1^ [27]. The control followed the same protocol without any stretch.

### Human model: Antioxidant supplementation experiment using human subjects

#### Subjects and downhill eccentric running protocol

Twenty-two moderately physically active males (age: 18–35; VO_2max_: 45–65 mL•kg^-1^•min^-1^) were recruited for this study. Subjects who fit any of the following criteria were excluded from the study: smokers, musculoskeletal limitations, use of any medication that may alter immune function and/or cardiovascular function including anti-inflammatory drugs, or were taking any antioxidant supplementation three months prior to the participation of the study. Qualified subjects were randomly assigned into a supplement (S) (age: 20.5 ± 0.7 yr; weight: 74.3 ± 3.2 kg; VO_2max_: 54.6 ± 1.0 mL•kg^-1^•min^-1^; *n* = 11) or placebo (P) (age: 21.3 ± 1.2 yr; weight: 75.2 ± 2.5 kg; VO_2max_: 53.1 ± 0.9 mL•kg^-1^•min^-1^; *n* = 11) group. The supplement (capsules) consisted of 1000 mg of vitamin C and 400 IU of vitamin E. The placebo (maltodextrin) was in capsules that were visually identical to the supplement. Supplementation (antioxidant or placebo) was administered daily for two weeks before each downhill running trial and two additional days after each trial in a double-blind manner. All participants provided written consent upon the explanation of the procedures and risks associated with this study. All consent form and study procedures were approved by Purdue University Institutional Review Boards (IRBs) charged with ethical review of proposed research with human subjects (protocol # 1005009339).

All participants completed two downhill running trials (1D and 2D) performed three weeks apart. All trials were performed in the morning following an overnight fast and refraining from strenuous exercise for 48 h. The trials consisted of a 3-min warm up followed by a 40-min downhill (-10% grade) run at a speed that elicited 65–70% VO_2max_. Three weeks later, subjects repeated the downhill run. In order to monitor the exercise intensity, heart rate, oxygen consumption, and rating of perceived exertion were recorded every 5 minutes during the exercise trials. Whole blood was drawn prior to each trial and at several time points POST. Total leukocyte count, leukocyte subset apoptosis, and total circulating leukocyte apoptotic-related proteins were measured. Supplementation compliance was confirmed by the plasma vitamin E status which was measured upon enrollment in the study (baseline), prior to 1D (PRE 1D) and prior to 2D (PRE 2D) for each subject.

#### Blood sample preparation and measurements

Four milliliters of whole blood were collected in an EDTA tube from an antecubital vein PRE immediately followed by exercise (0 h), and 6, 24, and 48 h post exercise. Dextran sedimentation followed by two rounds of hypotonic lysis was performed to remove erythrocytes and platelets during the preparation of peripheral blood leukocytes [28]. The blood sample was then briefly mixed with a 0.6% dextran solution (0.9% saline solute). The solution was set aside to sedimentate for 40 min, then approximately 1.5–2.0 mL supernatant was transferred to a 15 mL centrifuge tube and centrifuged at 200g for 10 min at 4°C. The resulting supernatant was extracted, and 9 mL of deionized water was added to the cell pellets. After 10 s, 1 mL of 10×PBS was added, mixed, and centrifuged at 200 g for another 10 min at 4°C. The procedure was repeated twice until red blood cells were no longer visible. Isolated leukocytes were re-suspended in 1 mL of PBS. 0.4% Trypan Blue stain (10 μL; Mediatech, Inc.) was added to 10 μL of the cell suspension, and the mixture (10 μL) was pipetted into a hemocytometer to collect the total WBC count. Cell viability was measured using light microscopy (Microscoptics IV 900 Series). The remaining mixture was centrifuged at 200 g for 10 min and the supernatant was decanted. Isolated leukocyte pellets were immediately stored at -80°C until analyzed for apoptotic proteins (Bcl-2 and Bax).

The quantification of vitamin E followed the protocol of Jiang *et al*. [29]. To each screw-top glass tube that was placed on ice, the following solution was added in sequence: methanol (2 ml), cold hexane (5 ml; stored with water layer), PBS (300 μL), butylated hydroxytoluene (BHT; 20 μl; 46 mg BHT in 1 mL methanol), and plasma sample (100 μL). Tubes were capped and vortexed for 1 min, then centrifuged at 3000 rpm at 4°C for 5 min. Four ml of the upper hexane layer was removed to 13×100 glass tubes and evaporated under nitrogen for at least 30 min. Later, 200 μL of ethanol was added to the dried hexane fraction, then wells was rinsed, and the mixture was transferred to 0.5 mL Eppendorf safety-lock tubes and stored at -80°C until analysis. Additionally, vitamin C was separated on a 150 × 4.6 mm, 5 μm Supelcosil LC18-DB column and eluted by 95:5 (v/v) methanol-0.1 M lithium acetate (25 mM, pH 4.75, flow rate of 1.3 mL/min). α-tocopherol was measured using HPLC with electrochemical detection (Model 5200A Coulochem II; ESA, Inc., Chemlford, MA) and a Model 5011 high sensitivity analytical cell. Data was extracted using TotalChrom Navigator software. Intra- and inter-assay of the coefficient of variation (CV) were 9.0% and 2.2%, respectively.

#### Apoptotic protein measurements

Bax and Bcl-2 in leukocytes were measured using an ELISA technique in accordance with the manufacturer's protocol (DuoSet, R&D Systems, Inc. Minneapolis, MN). Briefly, after cells were solubilized on ice at 1×107 cells/mL in lysis buffer for 15 min, samples were centrifuged at 2000 × g for 5 min. The supernatant was collected for analysis. Plates were prepared as follows: the capture antibody was diluted to a working concentration of 2.0 μg/mL in PBS for Bax (mouse anti-human Bax-α antibody) and 4 μg/mL in PBS for Bcl-2 (mouse anti-human Bcl-2 antibody). Plates were pre-coated with 100 μl per well of the diluted antibody, then sealed and incubated at room temperate overnight. Each well was aspirated and washed with 400 μL of wash buffer (0.05% Tween 20 in PBS, pH 7.3) three times and then blocked with 300 μL block buffer (1% bovine serum albumin (BSA), 0.05% NaN3, in PBS, pH 7.3). Plates were incubated at room temperature for 2 h. Before adding the sample, block buffer was removed by aspiration and washed three times as previously described. Then 100 μL of sample or standards in IC Diluent (1 mM EDTA, 0.5% Triton X-100 in PBS, pH = 7.3) were added to each well. Plates were then covered by a plate sealer and incubated for 2h at room temperature. Then the aspiration and wash procedure were repeated. The detection antibody (biotinylated mouse anti-human Bax-α or Bcl-2 antibody) was diluted to a working concentration of 125 ng/mL in IC Diluent (1% BSA in PBS, pH 7.3) for Bax and 0.5 μg/ml for Bcl-2. 100 μL of detection antibody was added to each well. Plates were covered with plate sealers and incubated for 2 h at room temperature.

Immediately before analysis, streptavidin-HRP was diluted to a working concentration using IC Diluent and 100 μL of the diluted streptavidin-HRP was added to each well. Plates were incubated for 20 min at room temperature in the dark, followed by three washes. Then, 100 μL of substrate solution was added to each well, and the plates were incubated for 20 min at room temperature without direct light, and 50 μL of stop solution was added to each well. A microplate reader (Model ELx 800, Bio- Tek Instruments, Inc. VT, USA) was used to determine the optical density of each well by subtracting the readings at 540 nm from the readings at 450 nm. Each sample was prepared in duplicate. Intra- and inter-assay CV for Bcl-2 concentration were 6.3% and 5.0%, respectively; and 7.3% and 6.9%, respectively for Bax concentration.

#### Morphological identification of leukocyte apoptosis with modified Giemsa

Five microliters of fresh whole blood were pipetted onto glass microscope slides (VWR Scientific, PA, USA). The slides were placed in undiluted Giemsa stain (Modified Giemsa, Sigma-Aldrich, MO, USA) for 2 min after being air dried and were then submerged in deionized water for 3 min. Slides were then rinsed in deionized water and allowed to air dry before evaluation. Two slides were prepared for each sample. Each blood film was evaluated under a light microscope (Micromaster; Fisher Scientific, PA, USA) in a blinded fashion. One hundred cells including leukocytes, lymphocytes, and neutrophils were counted per slide for apoptotic evaluation. The apoptotic index (AI; %) was determined by the number of apoptotic cells over 100 respective cells that were randomly selected. Each sample was counted in duplicates and AI was recorded as the average of two slides. If counts varied by more than 5%, the slides in question were recounted [30].

#### Statistics

The student’s *t* test was used to analyze the mouse data for statistical significance between the control and stretched muscles as well as to compare ROS data with and without SOD treatments via IBM SPSS software. The human data was analyzed using a mixed model ANOVA with repeated measures followed by adjusted Tukey’s post hoc tests using SAS (9.3). All values were presented as mean ± SE (IBM SPSS Statistics 21). *p* < 0.05 was considered statistically significant.

## Results and discussion

### Results

#### Diaphragmatic muscle force under stretch

**Fig 1:**
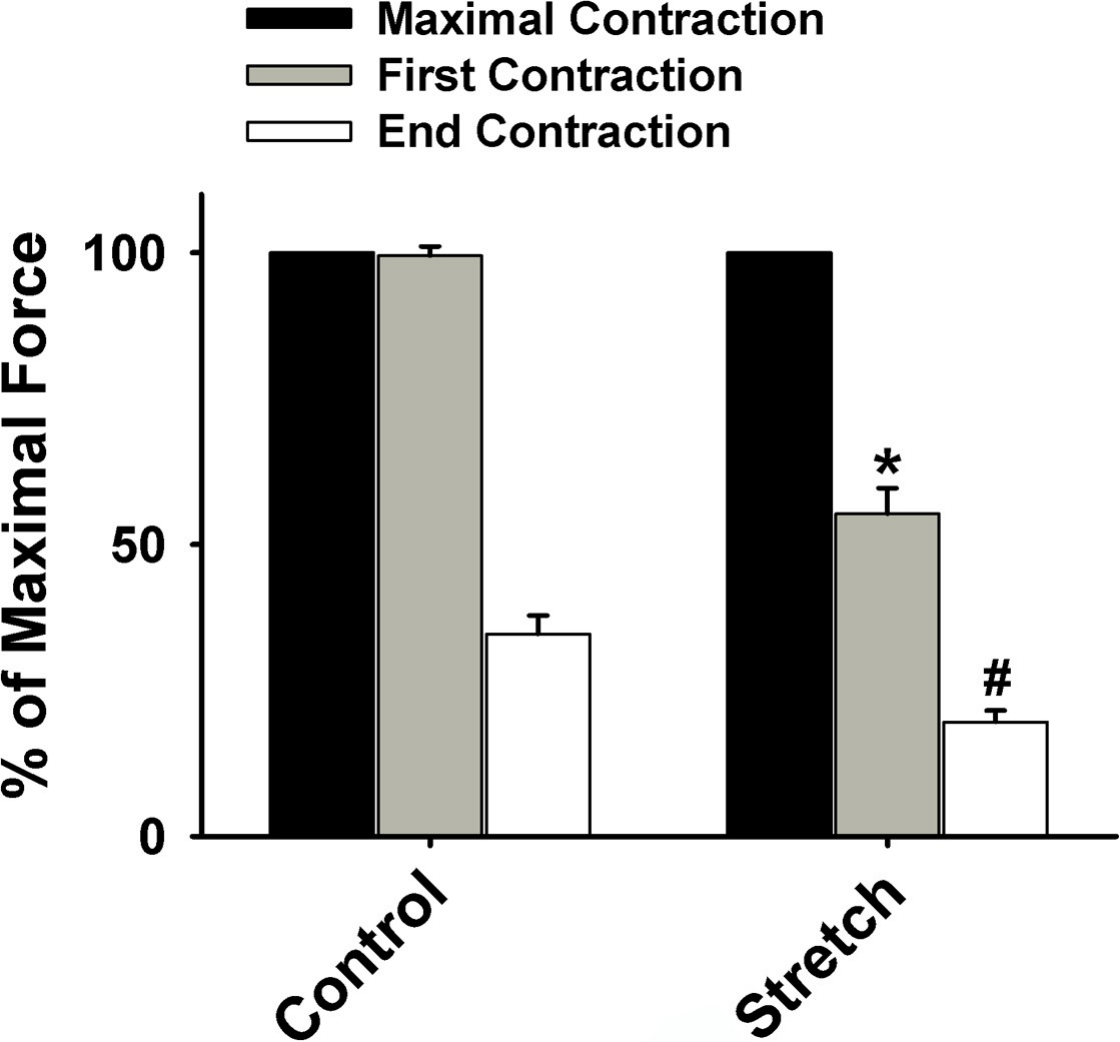
Grouped data showing the decline in diaphragmatic force (fatigue) from control (*n* = 13) and stretch (*n* = 10). Muscle contractile force was normalized by the maximal force during the baseline of each treatment group prior to stretch. *Significantly different from the force of the first contraction of control (*p* < 0.05). ^#^Significantly different from the force of the end contraction of control (*p* < 0.05).

**Fig 2:**
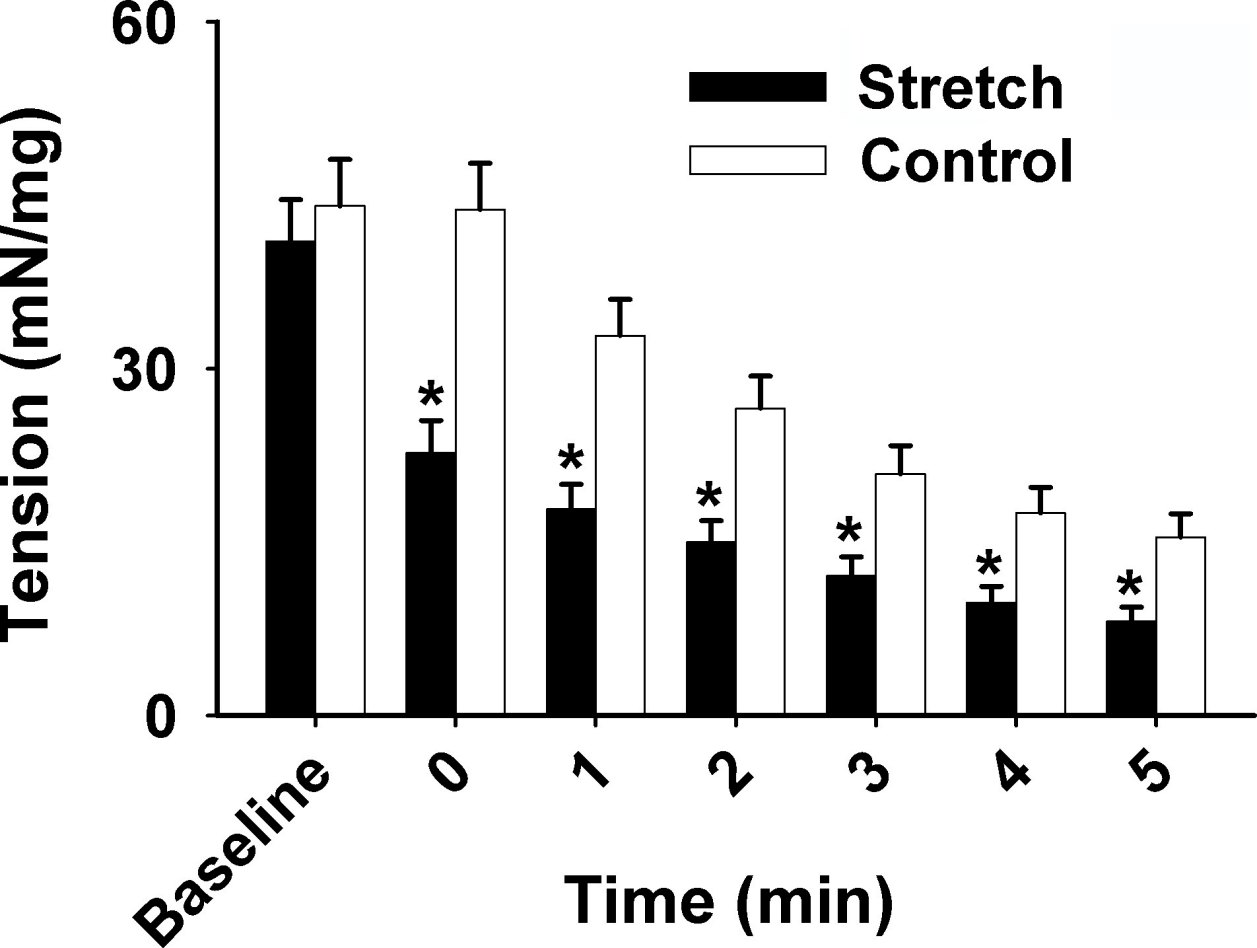
Grouped data showing the tension development (mN/mg) at baseline (the maximal force prior to tetanic contraction during equilibrium) and 0–5 min during the 5-min contractile period from the muscles at stretch (n = 10) *vs*. control (optimal length, *n* = 13). *Significantly difference from control (*p* < 0.05).

**Fig 3:**
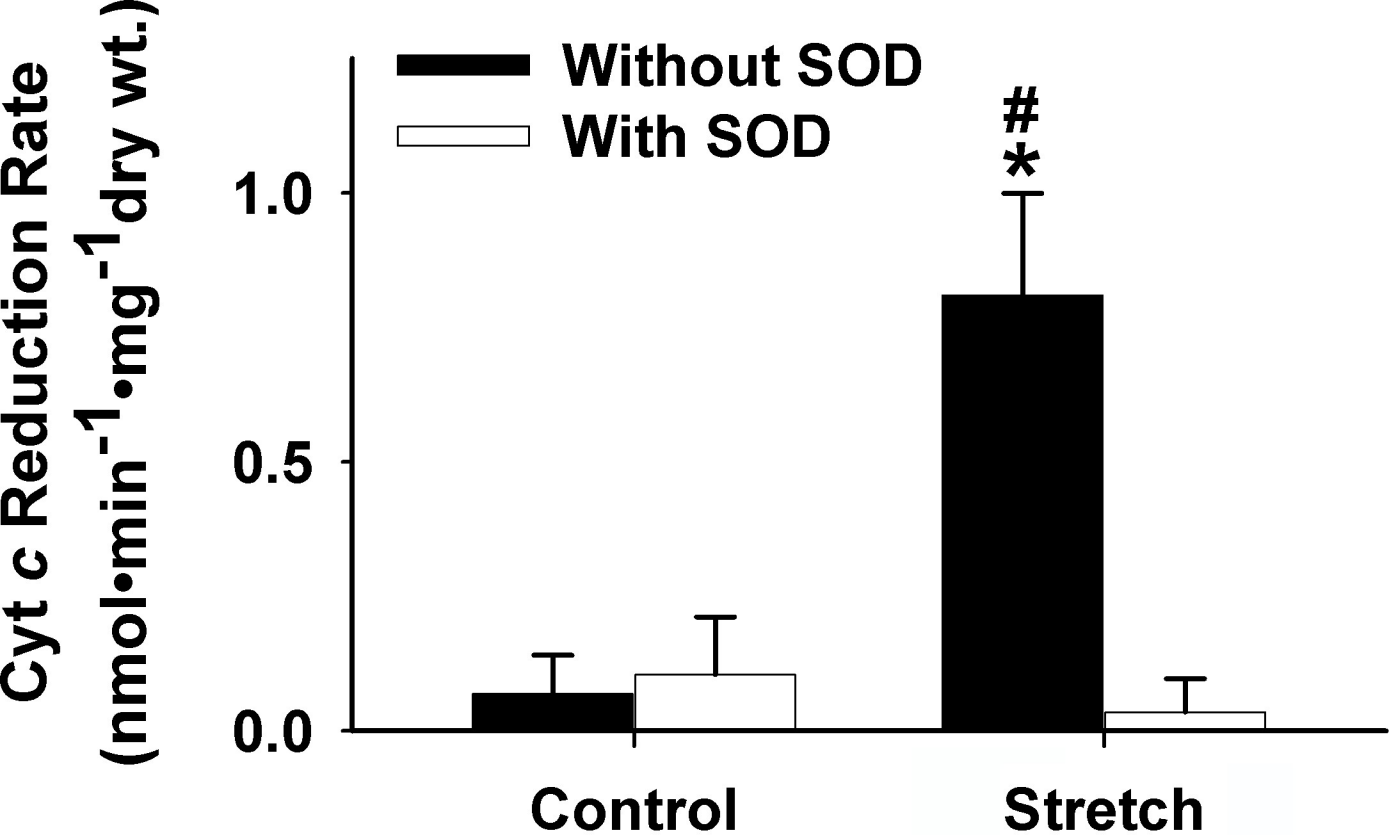
Grouped data showing the cyt *c* reduction rate (nmol•min^-1^•mg^-1^ dry wt) during the 5-min contractile period from the muscles of control (optimal length, *n* = 6), control + SOD (*n* = 10), stretch (*n* = 10), and stretch + SOD (*n* = 5). *Significant difference from control; ^#^Significant difference from SOD group of the same treatment (*p* < 0.05). cyt *c*, cytochrome *c*.

The percentage of diaphragmatic force at both first and end contraction in stretched muscle (*n* = 10) was significantly reduced in comparison to muscle with optimal length (*n* = 13; *p* < 0.05; Fig 1). Specifically, the development of muscle tension (mN/mg) in the stretched group (*n* = 10) was markedly lower than the control group (*n* = 13) at each subsequent time point (0–5 min) during the 5-min contractile period, suggesting that the stretching results in muscle force decline (*p* < 0.05; Fig 2). In addition, as shown in Fig 3, a significant elevation of cytochrome *c* (cyt *c*) reduction rate was observed in the stretched group (0.81 ± 0.18 nmol•min-1•mg^-1^ dry wt *vs*. 0.068 ± 0.072 nmol•min-1•mg^-1^ dry wt in control) and the application of superoxide dismutase (SOD) diminished this increase.

#### Compliance of supplementation

**Fig 4:**
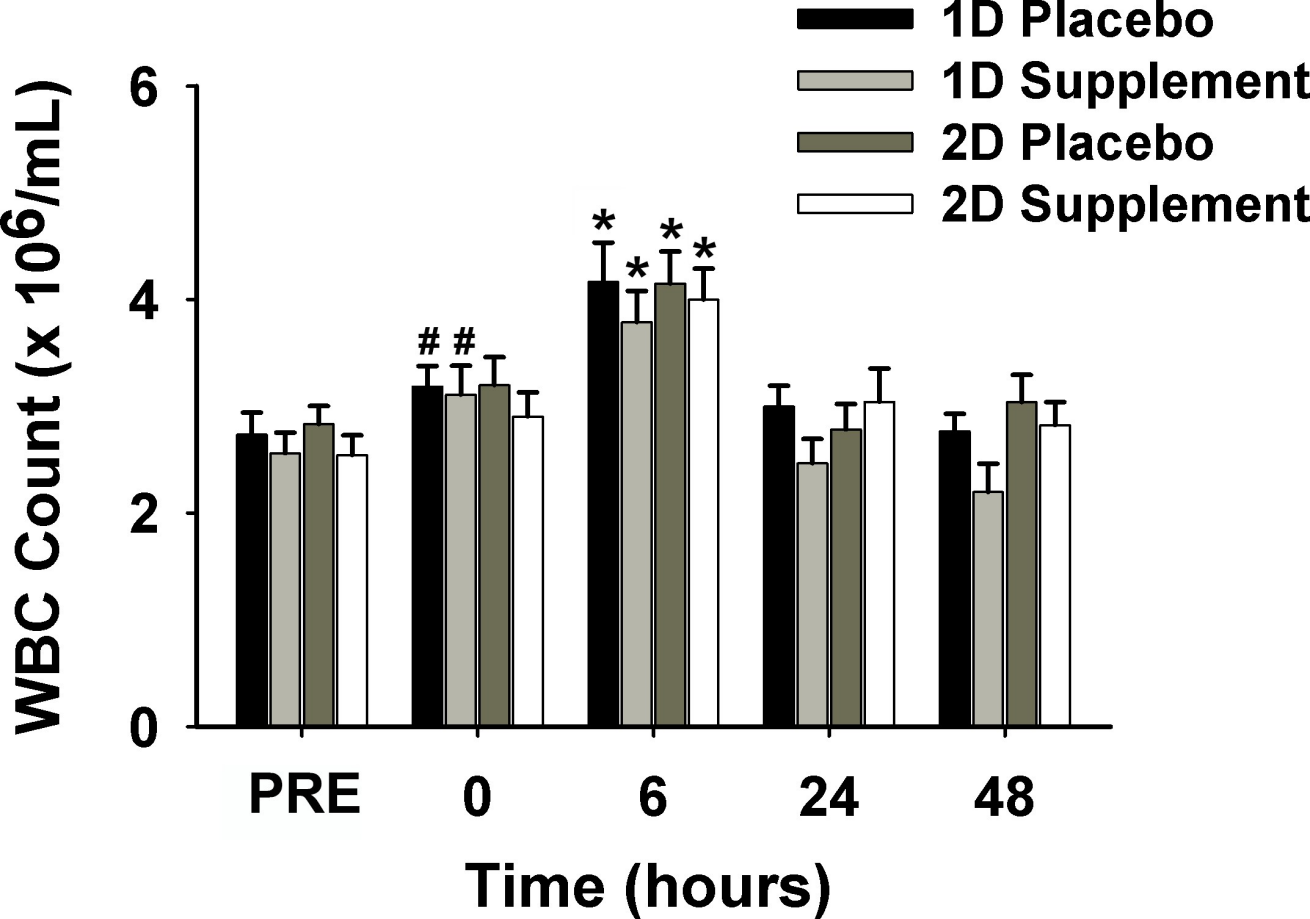
WBC count before exercise and immediately (0), 6, 24, and 48 h post-exercise in 1D and 2D. *Significantly greater than all other time points (*p* < 0.01); ^#^Significantly greater than PRE and 48 h post-exercise 1D (*p* < 0.05). 1D, first downhill run; 2D, second downhill run; PRE, before exercise; WBC, white blood cell.

Plasma α-tocopherol (vitamin E) status is shown in Table 1. Within the S group, plasma vitamin E was significantly higher prior to exercise (PRE) 2D than at baseline or PRE 1D (*p* < 0.01), and there an increase trend in plasma vitamin E from baseline to PRE 1D (*p* = 0.07). In Fig 4, white blood cell (WBC) count was significantly higher 6 h post-exercise (POST) (4.03 ± 0.30 ×106/mL) than at PRE (2.67 ± 0.18), 0 h (3.10 ± 0.23), 24 h (2.82±0.24) and 48 h (2.71±0.23) POST (*p* < 0.01).

**Table 1:** Plasma α-tocopherol concentration (μM) at baseline, PRE 1D and PRE 2D in the supplement (S) and placebo (P) groups.

Time	Group (*n* = 11/group)	
	S	P
Baseline	17.1 ± 1.4	20.2 ± 1.8
PRE 1D	26.1 ± 2.8	20.1 ± 3.0
PRE 2D	41.4 ± 3.7^#^	17.8 ± 1.8

Values are means ± SE

^#^Significantly higher than P; 1D, first downhill run; 2D, second downhill run

#### WBC count after downhill running

Additionally, an analysis with time effects suggests that WBC count at 0 h POST (3.15 ± 0.23) was significantly higher (*p*< 0.05) than PRE (2.65 ± 0.19) and 48 h POST (2.48 ± 0.23) following 1D (Fig 4). There were no supplementation effects on WBC count.

#### Peripheral leukocyte apoptosis-related proteins

**Fig 5:**
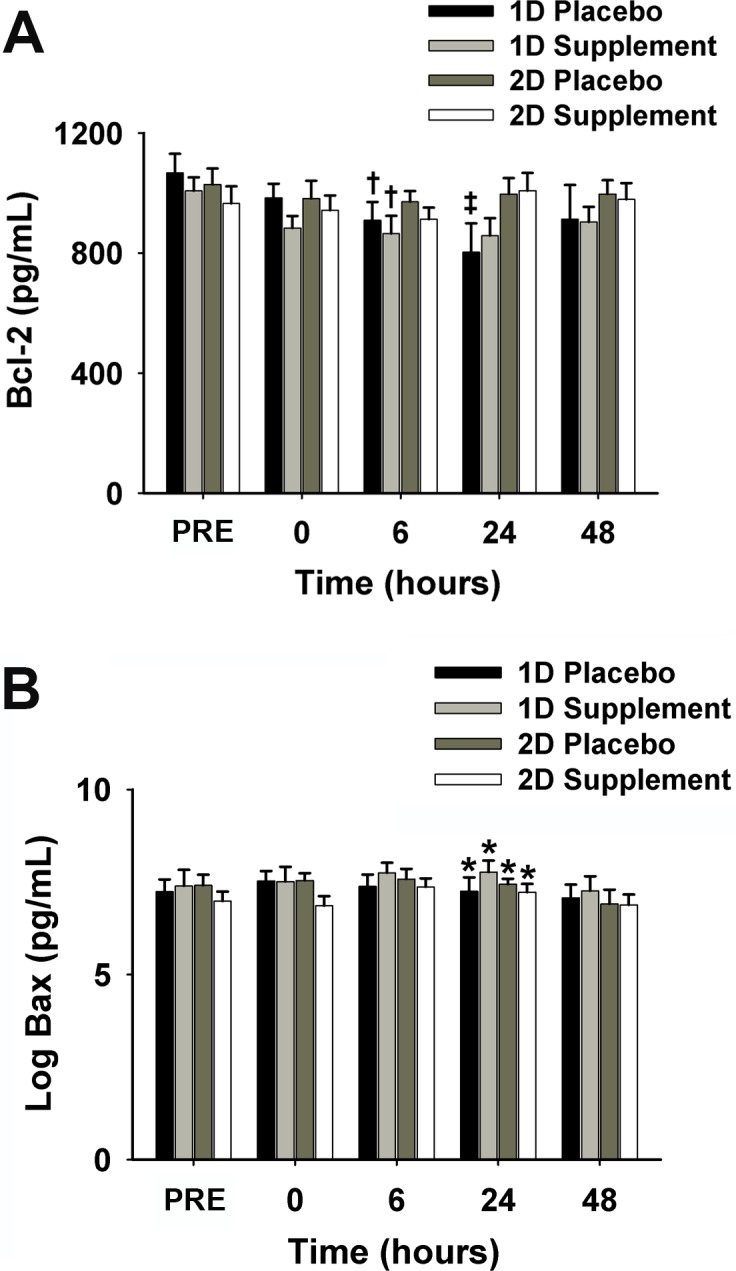
Bcl-2 concentration (A) and Log transformed Bax (B) before exercise and immediately (0), 6, 24, and 48 h post-exercise in 1D and 2D. ^†^Significantly lower than PRE following 1D (*p* < 0.05); ^‡^Significantly lower than PRE following 1D in P (*p* < 0.05); *Significantly higher than 48 h post-exercise (*p* < 0.01). 1D, first downhill run; 2D, second downhill run; S, supplement group; P, placebo group; PRE, before exercise.

As shown in Fig 5A, analysis of the trial (downhill running) effects indicates that B-cell lymphoma 2 (Bcl-2) following 2D (981±49 pg/mL) was significantly higher than 1D (927 ± 77 pg/mL), and was markedly lower at 6 h (918 ± 54 pg/mL) and 24 h (927 ± 74 pg/mL) POST than PRE (1022 ± 61 pg/mL) (*p* < 0.05). However, Bcl-2 concentration following 1D was significantly lower at 6 h POST compared to PRE (890 ± 67 pg/mL *vs*. 1052 ± 66 pg/mL; *p* < 0.05). Regarding supplement effects, Bcl-2 concentration at 24 h POST 1D was lower than PRE (794 ± 95 pg/mL *vs*. 1067 ± 64 pg/mL; *p* < 0.05) in only the P group. Bcl-2-associated X protein (Bax) concentration (Fig 5B) was logarithmically transformed because the data were not normally distributed. Bax concentrations at 6 h (7.46 ± 0.27 pg/ml) and 24 h (7.39 ± 0.28 pg/ml) POST were significantly higher than at 48 h POST (6.97 ± 0.35 pg/ml) (*p* < 0.01). There was no supplementation effect on Bax concentration.

**Fig 6:**
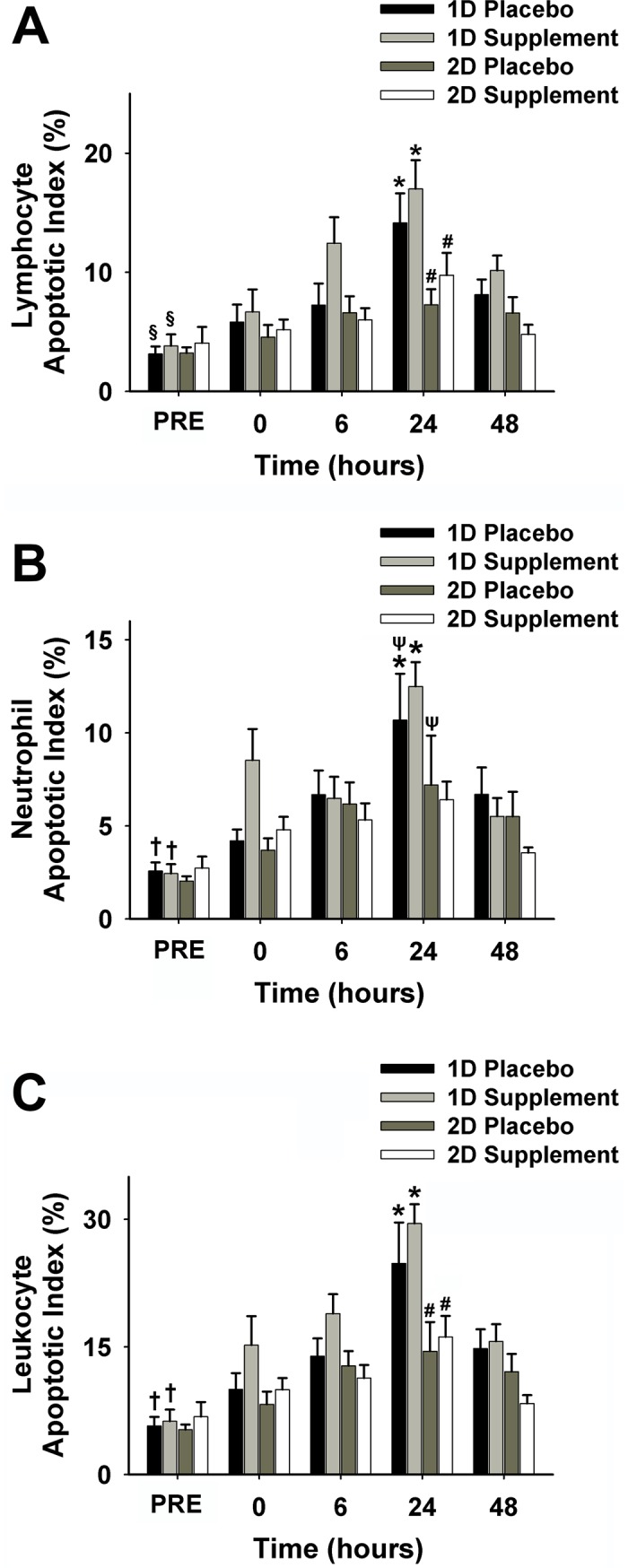
Apoptotic index for lymphocytes (A), neutrophils (B), and leukocytes (C) before exercise and immediately (0), 6, 24, and 48 h post-exercise in 1D and 2D. 2D significantly lower than 1D in (A) and (B) (*p* < 0.01). 2D supplement group significantly lower than 1D (C) (*p* <0.01); ^§^6, 24, and 48 h post-exercise were significantly higher than PRE (i.e. baseline) in 1D (*p* < 0.01); *24 h was significantly greater than 0, 6, and 48 h post-exercise in 1D (*p* < 0.01); ^#^Significantly higher than PRE in 2D (*p* < 0.01). ^ψ^ 24 h was significantly higher than PRE and 0 h post-exercise (*p* < 0.01); ^†^0, 6, 24, and 48 h post-exercise in 1D were significantly higher than PRE (*p* < 0.01). Note, 1D, first downhill run; 2D, second downhill run; PRE, before exercise.

Lymphocyte AI (Fig 6A) was lower following 2D (5.8 ±1.3%) than 1D (8.9 ± 2.1%) (*p* < 0.01). Within 1D, lymphocyte AI was higher at 6h, 24h, and 48h POST than PRE (*p* < 0.01), and values at 24h POST were higher than 0 h, 6 h, and 48 h POST (*p* < 0.01). Within 2D, lymphocyte AI at 24h post exercise was higher than PRE (*p* < 0.01). There were no supplementation effects on lymphocyte AI. Analysis of neutrophil AI is shown in Fig 6B and neutrophil AI was lower following 2D (4.7 ± 1.2%) than 1D (6.6 ±1.6%) (*p* < 0.01). Within 1D, neutrophil AI was higher at 0, 6, 24, and 48 h POST than PRE (*p* < 0.05) and reach peak at 24 h POST (*p* < 0.01). Following 2D, neutrophil AI was higher at 24 h POST than PRE and 0 h POST only in P (*p* < 0.01). Leukocyte Apoptotic Index (AI) (Fig 6C) was lower following 2D (10.5 ± 2.0%) than 1D (15.5 ± 3.5%) (*p* < 0.01). Within 1D, leukocyte AI was higher at 0 h, 6 h, 24 h, and 48 h POST than PRE (*p* < 0.01) and higher at 24 h POST than 0 h, 6 h and 48 h POST (*p* < 0.01). Within 2D, leukocyte AI at 24 h POST was higher than PRE (*p* < 0.01). There were no supplementation effects on leukocyte AI.

## Discussion

In this study, we observed increased extracellular O_2_^•–^in stretched muscle, which was associated with muscle force decline using a mouse model. This increase in ROS during stretching likely contributed to muscle damage related to eccentric contractions. In the related human study, combined supplementation of antioxidants ascorbic acid (vitamin C) and vitamin E resulted in decreased muscle soreness/muscle damage following downhill running [20]. Antioxidant supplementation appeared to enhance the adaptive immune response following a second bout of eccentrically-biased exercise, which is consistent with the repeated bout effect as shown by an attenuation of neutrophil apoptosis at 24h POST 2D.

Sarcomere lengthening has been shown to occur in repetitive eccentric contractions, potentially leading to rupture and muscle dysfunction [31]. The first part of the study evaluated the extracellular O_2_^•–^formation and associated muscle function during stretching. SOD is an effective O_2_^•–^scavenger, and it is therefore used to confirm the specificity of cyt *c* assay for O_2_^•–^[32]. Our results qualitatively indicated that extracellular O_2_^•–^release from the stretched muscle tissue was enhanced, which suggests that ROS can be generated during eccentric exercise from the stretched muscle, independent of the immune activation. O_2_^•–^is reported to contribute greatly to the modulation of skeletal muscle force production and fatigue [33], yet their effects under stretch-induced stress are not yet fully elucidated. Collectively, among several other undetermined factors, the release of extracellular O_2_^•–^likely plays a critical role in the decline of muscle force development during stretching. It is further suggested that stretching-induced sarcomere instability contributes to the initial stage of muscle damage in eccentric contractions [6]. The combined action of ROS increment and mechanical perturbation (e.g., mild or severe stretch) is associated with MAPK (ERK1/2) phosphorylation and the subsequent alteration of muscle cell differentiation [34].

Additional experiments are needed in order to conduct a thorough investigation of the potential interactions among ROS, muscle function under stretching, and eccentric contractions. For example, Liao *et al*. reported that ROS, mainly hydrogen peroxide (H_2_O_2_), stimulate the production of pro-inflammatory cytokine tumor necrosis factor alpha (TNF-α) via the nuclear factor kappa-light-chain-enhancer of activated B cells (NF-κB) pathway, which leads to inflammation and muscle proteolysis following eccentric contractions [35]. Inflammation promotes the infiltration of immune cells and further exacerbates ROS production through the process of oxidative burst [35, 36]. Increased oxidative stress and the associated elevation of leukocyte apoptosis have been implicated in muscle undergoing eccentric exercise [7].

The diaphragm is actually a mixture of both type I and type fibers, thus it is pretty similar to quadriceps muscles which are also a combination of both types of the fibers [37]. This is one of rationales to design our experiments in order to study the similarity of two muscles regarding the stretch-induced biological responses. Moreover, it is also easier to compare the two muscles, though they are located in different models.

Considering the potential detrimental effects of ROS related to stretching and eccentric contraction, the human portion of the study investigated whether antioxidant supplementation alleviates leukocyte apoptosis and apoptosis-related proteins following unaccustomed eccentrically-biased aerobic exercise. Although antioxidant supplementation did not attenuate the increase in total leukocyte AI following a downhill run, a significant elevation in neutrophil AI was observed at 24 h POST 2D in the P group but not in the S group, suggesting that supplementation exerted some protective effects on apoptotic states of the leukocyte subsets. Most researchers investigated apoptosis in lymphocytes [8, 38, 39] whereas only a few studied exercise-induced neutrophil apoptosis [11, 40]. Our study confirmed that unaccustomed eccentrically-biased exercise induces both lymphocyte and neutrophil apoptosis, both of which peaked at 24 h POST. This might partially explain the increased infection risk (e.g. upper respiratory tract infection) of athletes during the open window period following acute intensive exercise [41].

In addition, leukocytosis was evident immediately following 40 min of downhill running and WBCs were markedly elevated 2 h post downhill run (-10% grade; 50% VO_2max_) [42]. Such increase in circulating WBCs may be associated with acute inflammation following eccentric exercise-induced myocyte micro-structure disruption [43, 44]. Muscle damage-associated immune responses are characterized by the systemic release of leukocytes, leukocyte infiltration and elevated pro-inflammatory cytokines in damaged tissue [45]. However, other factors, such as epinephrine, that increases during exercise can also contribute to leukocytosis [46]. Thus, the exact cause of the WBC response to eccentrically-biased exercise is not fully elucidated. The timely resolution of inflammation has been attributed to programmed cell death of leukocytes. For example, neutrophils and lymphocytes may undergo apoptosis, which limits their capability to cause further inflammation [47]. We speculate that the increase in circulating WBCs may be associated with acute inflammation following eccentric exercise-induced myocyte micro-structure disruption [43, 44].

*In vitro* studies reported that ROS mediate apoptosis in various cell types [48–51]. Syu *et al*. conducted both *in vitro* and *in vivo* experiments and showed that exercise induced human neutrophil apoptosis by altering redox status in an intensity-dependent manner [11]. Acute exhaustive cycle ergometer exercise accelerated neutrophil apoptosis, which appeared to be mediated by increased ROS, whereas chronic moderate exercise (30 min a day, 5 days a week at 60% of maximal workload) attenuated neutrophil apoptosis by strengthening the antioxidant defense system (i.e., increased glutathione) [11]. Antioxidant administration including vitamin E, N-acetylcysteine (NAC), and other antioxidant combinations have been shown to prevent or inhibit cell apoptosis via redox-controlled mechanisms [52, 53]. For instance, NAC application completely prevents intensive and exhaustive exercise-induced T-lymphocyte apoptosis in mice spleens and bone marrow [54]. Inflammatory signals such as TNF and ROS associated with intensive exercise may promote cell apoptosis [55, 56]. Hartmann *et al*. demonstrated that vitamin E (1200 mg/day; 14 days prior to exercise) inhibited leukocyte DNA damage after a single bout of exhaustive exercise [57].

Moreover, ROS formation through mitochondria oxidative pathways, could evoke an inflammatory response following acute rigorous exercise promoting leukocyte apoptosis [38]. Despite the positive results of antioxidant use in scavenging excess ROS, the beneficial effect of vitamin C and E supplementation on exercise remains controversial [17]. Ristow *et al*. suggested that exogenous non-physiological doses of vitamin C and E may impair the favorable adaptations of regular exercise on healthy young men by blocking redox-sensitive transcription factors such as peroxisome proliferator-activated receptor gamma (PPARγ) and antioxidant enzymes such as SOD and glutathione peroxidase [58]. Unaccustomed eccentric exercise increases free radical generation, induces muscle damage, and results in impaired immune function, all of which are attenuated following a second bout of similar exercise [59, 60]. However, the mechanism of the adaptive immune response (e.g., less leukocyte apoptosis) as a result of repeated bouts of eccentrically-biased exercise, has not been explored.

In the present study, we observed that Bcl-2 concentration at 24 h POST was markedly lower than PRE in the P group but not in the S group following 1D. Antioxidant supplements appeared to attenuate the decrease in anti-apoptotic proteins, likely promoting cell survival. Consistent with our previous work [61], leukocyte apoptosis was significantly diminished following 2D. We found that antioxidant supplementation attenuated the exercise-induced increase in neutrophil apoptosis at 24 h POST 2D. It should also be noted that the decrease in Bcl-2 protein levels at 24 h POST 1D also was also blunted in the S group. This result could be expected by ROS tendency to upregulate pro-apoptotic protein expression [62]. These findings further support that ROS are involved in exercise-induced adaptations in the immune system.

Apoptosis can be triggered by intrinsic or extrinsic pathways. Intrinsic pathways arise from the mitochondria involving the Bcl-2 protein family as a central regulator [63]. Specifically, Bcl-2, Bcl-X, and Mcl-1 are anti-apoptotic proteins whereas Bax, Bcl-2-associated death promoter (Bad), and BH3 interacting-domain death agonist (Bid) are pro-apoptotic proteins. Bcl-2 protein is located at intracellular membranes and plays an important role in maintaining mitochondrial membrane integrity by preventing the release of cyt *c* into the cytosol, subsequently activating caspase-9 and the downstream cell apoptotic cascade. Although a reduction in Bcl-2 might increase cell susceptibility to apoptosis, it may not necessarily induce apoptosis without an additional external stressor [64]. Bax also belongs to the Bcl-2 protein family but has a pro-apoptotic role. Translocation of Bax into the mitochondrial outer membrane increases membrane permeability which allows for the release of pro-apoptotic factors (e.g., cyt *c*). Furthermore, apoptosis occurs in a dynamic and transient manner and once an apoptotic body is formed, it is immediately removed by phagocytosis. The lack of observed congruency between Bcl-2 concentration and leukocyte apoptosis in the present study is not novel. Krüger *et al*. also found that lymphocyte Bcl-2 content was significantly lowered 24 h after intensive resistance exercise but without an increase in lymphocyte apoptosis [8].

The current study showed that an eccentric downhill run elicited an immune response (such as leukocytosis and leukocyte apoptosis). A second downhill run and antioxidant supplementation attenuated leukocyte apoptosis and muscle soreness/muscle damage, which was consistent with findings of our previous work [20]. Eccentric-induced impaired muscle function has been associated with mechanical damage, including sarcomere disruption and local inflammatory responses, as demonstrated by delayed muscle soreness [6]. Interestingly, Close *et al*. observed an increase in ROS, as measured by electron spin resonance (ESR), only after the peak muscle function decline and delayed onset of muscle soreness which followed eccentric downhill running in human subjects. Therefore, it has been suggested that ROS are not major contributors to muscle damage in eccentric exercise [65]. However, the exact source of ROS in lengthening exercise has not been fully investigated in human studies. By monitoring the ROS formation in skeletal muscle *ex vivo*, the data from current studies showed a marked release of O_2_^•–^from stretched muscle during contraction.

The inconsistencies found between the animal and human studies may be due to the differences in contractile protocol and the methods used for detecting ROS. The animal stretching model is aimed to further explore the redox relations of the impaired muscle function following stretch preconditioning of the muscle. Although leukocytes have been suggested as a primary generator of ROS during inflammation [66], the *ex-vivo* experiments indicated that stretched skeletal muscle can produce ROS via other possible ROS sources during muscle contraction in the absence of leukocytes. There are many types of ROS generated from the biological system, including O_2_^•–^, H_2_O_2_, and hydroxyl radical (^•^OH). This study provided evidence that O_2_^•–^accounts for an important type of ROS formed during muscle lengthening activities. ROS are known to play multifaceted roles in mediating inflammatory responses including promoting neutrophil migration, killing microbes as well as inducing cell apoptosis [66]. Increased leukocyte apoptosis is a mechanism to terminate (or resolve) excessive inflammation [47]. However, the specific roles of excessive O_2_^•–^released from the skeletal muscle and whether they are main contributors to leukocyte apoptosis following eccentric exercise remain to be elucidated.

Sarcomere lengthening is considered a major mechanism underlying muscle damage associated with eccentric exercise [1]. Although it is generally accepted that oxidative stress can be induced during vigorous exercise, which is closely linked with immune activation [67, 68], the exact correlation between ROS formation and immune responses remains unclear in the context of eccentric training. Here we provided evidence that muscle lengthening can directly lead to prominent ROS production and force decline during contraction independent of immune cell activation. Antioxidants supplementation in human subjects appears to attenuate the decrease in anti-apoptotic protein but show no effect on the immune profiles following downhill running. Further study is needed to explore the correlation between ROS formation, immune activation and muscle dysfunction in eccentric exercise.

## Conclusions

In this study, we identified the involvement of extracellular ROS in stretching-induced muscle force decline. The increase in ROS during stretching likely contributes to muscle damages associated with eccentric contractions. In addition, oxidative stress may play a role in elevated leukocyte apoptosis following unaccustomed eccentric exercise, although other mediators may also contribute. Further, we observed an attenuation of leukocyte apoptosis following a second bout of eccentrically-biased exercise. Antioxidant supplementation appears to enhance this adaptive immune response by attenuating the decrease in anti-apoptotic proteins, perhaps via increasing antioxidant capacity. Additional research is required to elucidate the molecular mechanisms underlying muscle lengthening exercise in relation to redox alterations. With a multi-pronged strategy, we present our results in a novel dimension. Unlike previous studies, our approach particularly focuses on characterization of redox correlation in stretching-induced muscle force decline in both mice and human models. This allows us to more accurately determine its unique role in immune response during eccentric exercise.

## Supporting information

S1 Table Mouse cytochrome *c* data. (XLSX)

S2 Table Mouse muscle force data. (XLSX)

S3 Table Mouse muscle force data. (XLSX)

S4 Table Human raw data. (XLSX)

## References

[1] Hyldahl RD, Chen TC, Nosaka K. Mechanisms and Mediators of the Skeletal Muscle Repeated Bout Effect. Exerc Sport Sci Rev. 2017;45(1):24–33. 10.1249/JES.0000000000000095 27782911

[2] Philippou A, Halapas A, Maridaki M, Koutsilieris M. Type I insulin-like growth factor receptor signaling in skeletal muscle regeneration and hypertrophy. J Musculoskelet Neuronal Interact. 2007;7(3):208–18. 17947802

[3] Allen DG. Eccentric muscle damage: mechanisms of early reduction of force. Acta Physiol Scand. 2001;171(3):311–9. 10.1046/j.1365-201x.2001.00833.x 11412143

[4] Cheng W, Li B, Kajstura J, Li P, Wolin MS, Sonnenblick EH, et al Stretch-induced programmed myocyte cell death. J Clin Invest. 1995;96(5):2247–59. 10.1172/JCI118280 7593611 PMC185875

[5] Zollner AM, Abilez OJ, Bol M, Kuhl E. Stretching skeletal muscle: chronic muscle lengthening through sarcomerogenesis. PLoS One. 2012;7(10):e45661 10.1371/journal.pone.0045661 23049683 PMC3462200

[6] Proske U, Morgan DL. Muscle damage from eccentric exercise: mechanism, mechanical signs, adaptation and clinical applications. J Physiol. 2001;537(Pt 2):333–45. 10.1111/j.1469-7793.2001.00333.x 11731568 PMC2278966

[7] Park KS, Sedlock DA, Navalta JW, Lee MG, Kim SH. Leukocyte apoptosis and pro-/anti-apoptotic proteins following downhill running. Eur J Appl Physiol. 2011;111(9):2349–57. 10.1007/s00421-011-1907-2 21424274

[8] Kruger K, Agnischock S, Lechtermann A, Tiwari S, Mishra M, Pilat C, et al Intensive resistance exercise induces lymphocyte apoptosis via cortisol and glucocorticoid receptor-dependent pathways. J Appl Physiol (1985). 2011;110(5):1226–32. 10.1152/japplphysiol.01295.2010 21393471

[9] Tuan TC, Hsu TG, Fong MC, Hsu CF, Tsai KK, Lee CY, et al Deleterious effects of short-term, high-intensity exercise on immune function: evidence from leucocyte mitochondrial alterations and apoptosis. Br J Sports Med. 2008;42(1):11–5. 10.1136/bjsm.2006.029314 17504785

[10] Wang JS, Huang YH. Effects of exercise intensity on lymphocyte apoptosis induced by oxidative stress in men. Eur J Appl Physiol. 2005;95(4):290–7. 10.1007/s00421-005-0005-8 16096840

[11] Syu GD, Chen HI, Jen CJ. Severe exercise and exercise training exert opposite effects on human neutrophil apoptosis via altering the redox status. PLoS One. 2011;6(9):e24385 10.1371/journal.pone.0024385 21931703 PMC3170310

[12] Scheel-Toellner D, Wang K, Assi LK, Webb PR, Craddock RM, Salmon M, et al Clustering of death receptors in lipid rafts initiates neutrophil spontaneous apoptosis. Biochem Soc Trans. 2004;32(Pt 5):679–81. 10.1042/BST0320679 15493986

[13] Zuo L, Zhou T, Pannell BK, Ziegler AC, Best TM. Biological and physiological role of reactive oxygen species—the good, the bad and the ugly. Acta Physiol (Oxf). 2015;214(3):329–48. 10.1111/apha.12515 25912260

[14] Powers SK, Duarte J, Kavazis AN, Talbert EE. Reactive oxygen species are signalling molecules for skeletal muscle adaptation. Exp Physiol. 2010;95(1):1–9. 10.1113/expphysiol.2009.050526 19880534 PMC2906150

[15] Franchi MV, Reeves ND, Narici MV. Skeletal Muscle Remodeling in Response to Eccentric vs. Concentric Loading: Morphological, Molecular, and Metabolic Adaptations. Front Physiol. 2017;8:447 10.3389/fphys.2017.00447 28725197 PMC5495834

[16] Maruhashi Y, Kitaoka K, Yoshiki Y, Nakamura R, Okano A, Nakamura K, et al ROS scavenging activity and muscle damage prevention in eccentric exercise in rats. J Physiol Sci. 2007;57(4):211–6. 10.2170/physiolsci.RP013006 17594755

[17] He F, Li J, Liu Z, Chuang CC, Yang W, Zuo L. Redox Mechanism of Reactive Oxygen Species in Exercise. Front Physiol. 2016;7:486 10.3389/fphys.2016.00486 27872595 PMC5097959

[18] McHugh MP. Recent advances in the understanding of the repeated bout effect: the protective effect against muscle damage from a single bout of eccentric exercise. Scand J Med Sci Sports. 2003;13(2):88–97. 10.1034/j.1600-0838.2003.02477.x 12641640

[19] Brooks SV, Vasilaki A, Larkin LM, McArdle A, Jackson MJ. Repeated bouts of aerobic exercise lead to reductions in skeletal muscle free radical generation and nuclear factor kappaB activation. J Physiol. 2008;586(16):3979–90. 10.1113/jphysiol.2008.155382 18591188 PMC2538922

[20] He F, Hockemeyer JAK, Sedlock D. Does Combined Antioxidant Vitamin Supplementation Blunt Repeated Bout Effect? Int J Sports Med. 2015;36(5):407–13. 10.1055/s-0034-1395630 25607519

[21] McHuge MP. Recent advances in the understanding of the repeated bout effect: theprotective effect against muscle damage from a single bout of eccentric exercise. Scand J Med Sci Sports. 2003;13:88–97. 12641640 10.1034/j.1600-0838.2003.02477.x

[22] Hody S, Lacrosse Z, Leprince P, Collodoro M, Croisier JL, Rogister B. Effects of eccentrically and concentrically biased training on mouse muscle phenotype. Med Sci Sports Exerc. 2013;45(8):1460–8. 10.1249/MSS.0b013e3182894a33 23439418

[23] Zuo L, Diaz PT, Chien MT, Roberts WJ, Kishek J, Best TM, et al PO2 cycling reduces diaphragm fatigue by attenuating ROS formation. PLoS One. 2014;9(10):e109884 10.1371/journal.pone.0109884 25299212 PMC4192541

[24] Zuo L, Hallman AH, Roberts WJ, Wagner PD, Hogan MC. Superoxide release from contracting skeletal muscle in pulmonary TNF-alpha overexpression mice. Am J Physiol Regul Integr Comp Physiol. 2014;306(1):R75–81. 10.1152/ajpregu.00425.2013 24196666 PMC3921307

[25] Zuo L, Nogueira L, Hogan MC. Effect of pulmonary TNF-alpha overexpression on mouse isolated skeletal muscle function. American Journal of Physiology-Regulatory Integrative and Comparative Physiology. 2011;301(4):R1025–R31. 10.1152/ajpregu.00126.2011 21697519 PMC3197448

[26] Zuo L, Pannell BK, Re AT, Best TM, Wagner PD. Po2 cycling protects diaphragm function during reoxygenation via ROS, Akt, ERK, and mitochondrial channels. Am J Physiol Cell Physiol. 2015;309(11):C759–66. 10.1152/ajpcell.00174.2015 26423578

[27] Kolbeck RC, She ZW, Callahan LA, Nosek TM. Increased superoxide production during fatigue in the perfused rat diaphragm. Am J Respir Crit Care Med. 1997;156(1):140–5. 10.1164/ajrccm.156.1.9610041 9230738

[28] Heit B, Liu L, Colarusso P, Puri KD, Kubes P. PI3K accelerates, but is not required for, neutrophil chemotaxis to fMLP. J Cell Sci. 2008;121(Pt 2):205–14. 10.1242/jcs.020412 18187452

[29] Jiang Q, Freiser H, Wood KV, Yin X. Identification and quantitation of novel vitamin E metabolites, sulfated long-chain carboxychromanols, in human A549 cells and in rats. J Lipid Res. 2007;48(5):1221–30. 10.1194/jlr.D700001-JLR200 17299205 PMC2185712

[30] Navalta JW, McFarlin BK, Lyons S, Arnett SW, Schafer MA. Cognitive awareness of carbohydrate intake does not alter exercise-induced lymphocyte apoptosis. Clinics (Sao Paulo). 2011;66(2):197–202. 21484033 10.1590/S1807-59322011000200003 PMC3059873

[31] Margaritelis NV, Theodorou AA, Baltzopoulos V, Maganaris CN, Paschalis V, Kyparos A, et al Muscle damage and inflammation after eccentric exercise: can the repeated bout effect be removed? Physiol Rep. 2015;3(12). 10.14814/phy2.12648 26660557 PMC4760450

[32] Zuo L, Christofi FL, Wright VP, Liu CY, Merola AJ, Berliner LJ, et al Intra- and extracellular measurement of reactive oxygen species produced during heat stress in diaphragm muscle. Am J Physiol Cell Physiol. 2000;279(4):C1058–66. 10.1152/ajpcell.2000.279.4.C1058 11003586

[33] Jackson MJ. Reactive oxygen species and redox-regulation of skeletal muscle adaptations to exercise. Philos Trans R Soc Lond B Biol Sci. 2005;360(1464):2285–91. 10.1098/rstb.2005.1773 16321798 PMC1569586

[34] Wretman C, Lionikas A, Widegren U, Lannergren J, Westerblad H, Henriksson J. Effects of concentric and eccentric contractions on phosphorylation of MAPK(erk1/2) and MAPK(p38) in isolated rat skeletal muscle. J Physiol. 2001;535(Pt 1):155–64. 10.1111/j.1469-7793.2001.00155.x 11507166 PMC2278759

[35] Liao P, Zhou J, Ji LL, Zhang Y. Eccentric contraction induces inflammatory responses in rat skeletal muscle: role of tumor necrosis factor-alpha. Am J Physiol Regul Integr Comp Physiol. 2010;298(3):R599–607. 10.1152/ajpregu.00480.2009 20007518

[36] Silva LA, Bom KF, Tromm CB, Rosa GL, Mariano I, Pozzi BG, et al Effect of eccentric training on mitochondrial function and oxidative stress in the skeletal muscle of rats. Braz J Med Biol Res. 2013;46(1):14–20. 10.1590/1414-431X20121956 23314343 PMC3854341

[37] Wijnhoven JH, Janssen AJ, van Kuppevelt TH, Rodenburg RJ, Dekhuijzen PN. Metabolic capacity of the diaphragm in patients with COPD. Respir Med. 2006;100(6):1064–71. 10.1016/j.rmed.2005.09.029 16257195

[38] Mooren FC, Lechtermann A, Volker K. Exercise-induced apoptosis of lymphocytes depends on training status. Med Sci Sports Exerc. 2004;36(9):1476–83. 15354026 10.1249/01.mss.0000139897.34521.e9

[39] Navalta JW, Sedlock DA, Park KS. Blood treatment influences the yield of apoptotic lymphocytes after maximal exercise. Med Sci Sports Exerc. 2005;37(3):369–73. 15741833 10.1249/01.mss.0000155433.08698.c1

[40] Lagranha CJ, Senna SM, de Lima TM, Silva E, Doi SQ, Curi R, et al Beneficial effect of glutamine on exercise-induced apoptosis of rat neutrophils. Med Sci Sports Exerc. 2004;36(2):210–7. 10.1249/01.MSS.0000113490.98089.B1 14767242

[41] Moreira A, Delgado L, Moreira P, Haahtela T. Does exercise increase the risk of upper respiratory tract infections? Br Med Bull. 2009;90:111–31. 10.1093/bmb/ldp010 19336500

[42] Smith LL, McCammon M, Smith S, Chamness M, Israel RG, O'Brien KF. White blood cell response to uphill walking and downhill jogging at similar metabolic loads. Eur J Appl Physiol Occup Physiol. 1989;58(8):833–7. 2767064 10.1007/BF02332215

[43] Petersen EW, Ostrowski K, Ibfelt T, Richelle M, Offord E, Halkjaer-Kristensen J, et al Effect of vitamin supplementation on cytokine response and on muscle damage after strenuous exercise. Am J Physiol Cell Physiol. 2001;280(6):C1570–5. 10.1152/ajpcell.2001.280.6.C1570 11350752

[44] Pizza FX, Mitchell JB, Davis BH, Starling RD, Holtz RW, Bigelow N. Exercise-induced muscle damage: effect on circulating leukocyte and lymphocyte subsets. Med Sci Sports Exerc. 1995;27(3):363–70. 7752863

[45] Peake J, Nosaka K, Suzuki K. Characterization of inflammatory responses to eccentric exercise in humans. Exerc Immunol Rev. 2005;11:64–85. 16385845

[46] Bedoui S, Lechner S, Gebhardt T, Nave H, Beck-Sickinger AG, Straub RH, et al NPY modulates epinephrine-induced leukocytosis via Y-1 and Y-5 receptor activation in vivo: sympathetic co-transmission during leukocyte mobilization. J Neuroimmunol. 2002;132(1–2):25–33. 12417430 10.1016/s0165-5728(02)00278-3

[47] Savill J. Apoptosis in resolution of inflammation. J Leukoc Biol. 1997;61(4):375–80. 9103222 10.1002/jlb.61.4.375

[48] Kayan M, Naziroglu M, Ovey IS, Aykur M, Uguz AC, Yurekli VA. Non-Ionic Contrast Media Induces Oxidative Stress and Apoptosis Through Ca2+ Influx in Human Neutrophils. J Membr Biol. 2012;245(12):833–40. 10.1007/s00232-012-9491-x 22903554

[49] Zhang D, Zhou T, He F, Rong Y, Lee SH, Wu S, et al Reactive oxygen species formation and bystander effects in gradient irradiation on human breast cancer cells. Oncotarget. 2016;7(27):41622–36. 10.18632/oncotarget.9517 27223435 PMC5173083

[50] Espino J, Bejarano I, Paredes SD, Barriga C, Rodriguez AB, Pariente JA. Protective effect of melatonin against human leukocyte apoptosis induced by intracellular calcium overload: relation with its antioxidant actions. J Pineal Res. 2011;51(2):195–206. 10.1111/j.1600-079X.2011.00876.x 21470303

[51] Lin YS, Kuo HL, Kuo CF, Wang ST, Yang BC, Chen HI. Antioxidant administration inhibits exercise-induced thymocyte apoptosis in rats. Med Sci Sports Exerc. 1999;31(11):1594–8. 10589862 10.1097/00005768-199911000-00015

[52] Sandstrom PA, Mannie MD, Buttke TM. Inhibition of activation-induced death in T cell hybridomas by thiol antioxidants: oxidative stress as a mediator of apoptosis. J Leukoc Biol. 1994;55(2):221–6. 7507967 10.1002/jlb.55.2.221

[53] Marsh SA, Laursen PB, Pat BK, Gobe GC, Coombes JS. Bcl-2 in endothelial cells is increased by vitamin E and alpha-lipoic acid supplementation but not exercise training. J Mol Cell Cardiol. 2005;38(3):445–51. 10.1016/j.yjmcc.2004.11.026 15733904

[54] Kruger K, Frost S, Most E, Volker K, Pallauf J, Mooren FC. Exercise affects tissue lymphocyte apoptosis via redox-sensitive and Fas-dependent signaling pathways. Am J Physiol Regul Integr Comp Physiol. 2009;296(5):R1518–27. 10.1152/ajpregu.90994.2008 19261913

[55] Moldoveanu AI, Shephard RJ, Shek PN. Exercise elevates plasma levels but not gene expression of IL-1 beta, IL-6, and TNF-alpha in blood mononuclear cells. J Appl Physiol. 2000;89(4):1499–504. 10.1152/jappl.2000.89.4.1499 11007588

[56] Phaneuf S, Leeuwenburgh C. Apoptosis and exercise. Med Sci Sports Exerc. 2001;33(3):393–6. 10.1097/00005768-200103000-00010 11252065

[57] Hartmann A, Niess AM, Grunert-Fuchs M, Poch B, Speit G. Vitamin E prevents exercise-induced DNA damage. Mutat Res. 1995;346(4):195–202. 7753111 10.1016/0165-7992(95)90035-7

[58] Ristow M, Zarse K, Oberbach A, Kloting N, Birringer M, Kiehntopf M, et al Antioxidants prevent health-promoting effects of physical exercise in humans. Proc Natl Acad Sci U S A. 2009;106(21):8665–70. 10.1073/pnas.0903485106 19433800 PMC2680430

[59] Nikolaidis MG, Kerksick CM, Lamprecht M, McAnulty SR. Does vitamin C and E supplementation impair the favorable adaptations of regular exercise? Oxid Med Cell Longev. 2012;2012:707941 10.1155/2012/707941 22928084 PMC3425865

[60] Nieman DC, Henson DA, McAnulty SR, McAnulty L, Swick NS, Utter AC, et al Influence of vitamin C supplementation on oxidative and immune changes after an ultramarathon. J Appl Physiol (1985). 2002;92(5):1970–7. 10.1152/japplphysiol.00961.2001 11960947

[61] Park KS, Sedlock DA, Navalta JW, Weith HL. Exercise-Induced Muscle Damage and Immune Cell Apoptosis. Med Sci Sports Exerc. 2006;38(5):S412–S. 10.1249/00005768-200605001-01736

[62] Portt L, Norman G, Clapp C, Greenwood M, Greenwood MT. Anti-apoptosis and cell survival: a review. Biochim Biophys Acta. 2011;1813(1):238–59. 10.1016/j.bbamcr.2010.10.010 20969895

[63] Elmore S. Apoptosis: a review of programmed cell death. Toxicol Pathol. 2007;35(4):495–516. 10.1080/01926230701320337 17562483 PMC2117903

[64] Begum-Haque S, Haque A, Kasper LH. Apoptosis in Toxoplasma gondii activated T cells: the role of IFNgamma in enhanced alteration of Bcl-2 expression and mitochondrial membrane potential. Microb Pathog. 2009;47(5):281–8. 10.1016/j.micpath.2009.09.004 19748565 PMC2771447

[65] Close GL, Ashton T, Cable T, Doran D, MacLaren DPM. Eccentric exercise, isokinetic muscle torque and delayed onset muscle soreness: the role of reactive oxygen species. Eur J Appl Physiol. 2004;91(5–6):615–21. 10.1007/s00421-003-1012-2 14685863

[66] Mittal M, Siddiqui MR, Tran K, Reddy SP, Malik AB. Reactive Oxygen Species in Inflammation and Tissue Injury. Antioxidants & Redox Signaling. 2014;20(7):1126–67. 10.1089/ars.2012.5149 23991888 PMC3929010

[67] Peake JM, Suzuki K, Coombes JS. The influence of antioxidant supplementation on markers of inflammation and the relationship to oxidative stress after exercise. J Nutr Biochem. 2007;18(6):357–71. 10.1016/j.jnutbio.2006.10.005 17156994

[68] Silva LA, Silveira PCL, Ronsani MM, Souza PS, Scheffer D, Vieira LC, et al Taurine supplementation decreases oxidative stress in skeletal muscle after eccentric exercise. Cell Biochem Funct. 2011;29(1):43–9. 10.1002/cbf.1716 21264889

