# Correction: Redox correlation in muscle lengthening and immune response in eccentric exercise

**DOI:** 10.1371/journal.pone.0211246

**Published:** 2019-01-17

**Authors:** Feng He, Chia-Chen Chuang, Tingyang Zhou, Qing Jiang, Darlene A. Sedlock, Li Zuo

In the Author Contributions section, Feng He (FH) should be listed as one of the persons who acquired funding.
